# Massively Parallel DNA Sequencing Facilitates Diagnosis of Patients with Usher Syndrome Type 1

**DOI:** 10.1371/journal.pone.0090688

**Published:** 2014-03-11

**Authors:** Hidekane Yoshimura, Satoshi Iwasaki, Shin-ya Nishio, Kozo Kumakawa, Tetsuya Tono, Yumiko Kobayashi, Hiroaki Sato, Kyoko Nagai, Kotaro Ishikawa, Tetsuo Ikezono, Yasushi Naito, Kunihiro Fukushima, Chie Oshikawa, Takashi Kimitsuki, Hiroshi Nakanishi, Shin-ichi Usami

**Affiliations:** 1 Department of Otorhinolaryngology, Shinshu University School of Medicine, Matsumoto, Nagano, Japan; 2 Department of Hearing Implant Sciences, Shinshu University School of Medicine, Matsumoto, Nagano, Japan; 3 Department of Otorhinolaryngology, International University of Health and Welfare, Mita Hospital, Minatoku, Tokyo, Japan; 4 Department of Otolaryngology, Okinaka Memorial Institute for Medical Research, Toranomon Hospital, Minatoku, Tokyo, Japan; 5 Department of Otorhinolaryngology-Head and Neck Surgery, Miyazaki University School of Medicine, Miyazaki, Miyazaki, Japan; 6 Department of Otolaryngology-Head and Neck Surgery, Iwate Medical University, Morioka, Iwate, Japan; 7 Department of Otolaryngology-Head and Neck Surgery, Gunma University Graduate School of Medicine, Maebashi, Gunma, Japan; 8 Department of Otolaryngology, National Rehabilitation Center for Persons with Disabilities, Tokorozawa, Saitama, Japan; 9 Department of Otolaryngology, Saitama Medical University, Moroyama, Saitama, Japan; 10 Department of Otolaryngology, Kobe City Medical Center General Hospital, Kobe, Hyougo, Japan; 11 Department of Otorhinolaryngology, Head and Neck Surgery, Okayama University Postgraduate School of Medicine, Dentistry and Pharmaceutical Science, Okayama, Okayama, Japan; 12 Department of Otorhinolaryngology, Graduate School of Medical Sciences, Kyushu University, Fukuoka, Fukuoka, Japan; 13 Department of Otorhinolaryngology, Kyushu Central Hospital, Fukuoka, Fukuoka, Japan; 14 Department of Otorhinolaryngology, Hamamatsu University School of Medicine, Hamamatsu, Shizuoka, Japan; Odense University hospital, Denmark

## Abstract

Usher syndrome is an autosomal recessive disorder manifesting hearing loss, retinitis pigmentosa and vestibular dysfunction, and having three clinical subtypes. Usher syndrome type 1 is the most severe subtype due to its profound hearing loss, lack of vestibular responses, and retinitis pigmentosa that appears in prepuberty. Six of the corresponding genes have been identified, making early diagnosis through DNA testing possible, with many immediate and several long-term advantages for patients and their families. However, the conventional genetic techniques, such as direct sequence analysis, are both time-consuming and expensive. Targeted exon sequencing of selected genes using the massively parallel DNA sequencing technology will potentially enable us to systematically tackle previously intractable monogenic disorders and improve molecular diagnosis.

Using this technique combined with direct sequence analysis, we screened 17 unrelated Usher syndrome type 1 patients and detected probable pathogenic variants in the 16 of them (94.1%) who carried at least one mutation. Seven patients had the *MYO7A* mutation (41.2%), which is the most common type in Japanese. Most of the mutations were detected by only the massively parallel DNA sequencing. We report here four patients, who had probable pathogenic mutations in two different Usher syndrome type 1 genes, and one case of *MYO7A*/*PCDH15* digenic inheritance.

This is the first report of Usher syndrome mutation analysis using massively parallel DNA sequencing and the frequency of Usher syndrome type 1 genes in Japanese. Mutation screening using this technique has the power to quickly identify mutations of many causative genes while maintaining cost-benefit performance. In addition, the simultaneous mutation analysis of large numbers of genes is useful for detecting mutations in different genes that are possibly disease modifiers or of digenic inheritance.

## Introduction

Usher syndrome (USH) is an autosomal recessive disorder characterized by hearing loss (HL), retinitis pigmentosa (RP) and vestibular dysfunction. Three clinical subtypes can be distinguished. USH type 1 (USH1) is the most severe among them because of profound HL, absent vestibular responses, and prepubertal onset RP. USH type 2 (USH2) is characterized by congenital moderate to severe HL, with a high-frequency sloping configuration. The vestibular function is normal and onset of RP is in the first or second decade. The onset of the visual symptoms such as night blindness in USH usually occurs several years later than in USH1. USH type 3 (USH3) is characterized by variable onset of progressive HL, variable onset of RP, and variable impairment of vestibular function (normal to absent) [1], [2].

To date, nine genetic loci for USH1(*USH1B-H*, *J*, and *K*) have been mapped to chromosomes 11q13.5, 11p15.1, 10q22.1, 21q21, 10q21-q22, 17q24-q25, 15q22-q23 (*USH1H* and *J*), and 10p11.21–q21.1 [2], [3], [4]. Six of the corresponding genes have been identified: the actin-based motor protein myosin VIIa (*MYO7A*, USH1B) [5]; two cadherin-related proteins, cadherin 23 (*CDH23*, USH1D) [6] and protocadherin 15 (*PCDH15*, USH1F) [7]; and two scaffold proteins, harmonin (*USH1C*) [8] and sans (*USH1G*) [9]; the Ca^2+^- and integrin-binding protein (*CIB2*, USH1J) [4]. In Caucasian USH1 patients, previous studies showed that mutations in *MYO7A*, *USH1C*, *CDH23*, *PCDH15*, and *USH1G*, were found in 39–55%, 7–14%, 7–35%, 7–11%, and 0–7%, respectively (the frequency of *CIB2* is still unknown) [10], [11], [12]. In Japanese, Nakanishi et al. showed that *MYO7A* and *CDH23* mutations are present in USH1 patients [13], however, the frequency is not yet known. In addition, mutations in three corresponding genes (usherin *USH2A* [14], G protein-coupled receptor 98; *GPR98* [15], and deafness, autosomal recessive 31; *DFNB31* [16]) have been reported so far in USH2, and USH3 is caused by mutations in the clarin 1 (*CLRN1*) [17] gene.

Comprehensive molecular diagnosis of USH has been hampered both by genetic heterogeneity and the large number of exons for most of the USH genes. The six USH1 genes collectively contain 180 coding exons [4], [9], [10] the three USH2 genes comprise 175 coding exons [15], [16], [18], and the USH3 gene has five coding exons [17]. In addition some of these genes are alternatively spliced ([4], [7], [8], [16], [17] and NCBI database: http://www.ncbi.nlm.nih.gov/nuccore/). Thus far, large-scale mutation screening has been performed using direct sequence analysis, but that is both time-consuming and expensive. We thought that targeted exon sequencing of selected genes using the Massively Parallel DNA Sequencing (MPS) technology would enable us to systematically tackle previously intractable monogenic disorders and improve molecular diagnosis.

Therefore, in this study, we have conducted genetic analysis using MPS-based genetic screening to find mutations in nine causative USH genes (except *CIB2*) in Japanese USH1 patients.

## Results

Mutation analysis of the nine USH genes in 17 unrelated USH1 patients revealed 19 different probable pathogenic variants, of which 14 were novel (Table 1).

**Table 1:** Possible pathogenic variants found in this study.

Gene	Mutation type	Nucleotide change	Amino acid change	exon/intron number	Domain	control (in 384 alleles)	SIFT Score	PolyPhen Score	Reference
*MYO7A*	Frameshift	c.1623dup	p.Lys542GlnfsX5	Exon 14	-	N/A	-	-	Le Quesne Stabej et al. (2012)
		c.4482_4483insTG	p.Trp1495CysfsX55	Exon 34	-	N/A	-	-	This study
		c.6205_6206delAT	p.Ile2069ProfsX6	Exon 45	-	N/A	-	-	This study
	Nonsense	c.1477C>T	p.Gln493X	Exon 13	-	N/A	-	-	This study
		c.1708C>T	p.Arg570X	Exon 15	-	N/A	-	-	This study
		c.2115C>A	p.Cys705X	Exon 18	-	N/A	-	-	This study
		c.6321G>A	p.Trp2107X	Exon 46	-	N/A	-	-	This study
	Missense	c.2074G>A	p.Val692Met	Exon 17	Motor domain	0	0.09	0.982	This study
		c.2311G>T	p.Ala771Ser	Exon 20	IQ 2	0.0026	0.01	0.825	Nakanishi et al. (2010)
		c.6028G>A	p.Asp2010Asn	Exon 44	FERM 2	0	0	0.925	Jacobson et al. (2009)
*CDH23*	Frameshift	c.3567delG	p.Arg1189ArgfsX5	Exon 30	-	N/A	-	-	This study
		c.5780_5781delCT	p.Ser1927Cysfs16	Exon 44	-	N/A	-	-	This study
	Splicing	c.5821-2A>G	?	Intron 44	-	N/A	-	-	This study
	Nonsense	c.6319C>T	p.Arg2107X	Exon 48	-	N/A	-	-	Nakanishi et al. (2010)
*PCDH15*	Splicing	c.158-1G>A	?	Intron 3	-	N/A	-	-	This study
	Nonsense	c.1006C>T	p.Arg336X	Exon 10	-	N/A	-	-	This study
		c.2971C>T	p.Arg991X	Exon 22	-	N/A	-	-	Roux et al. (2006)
		c.3337G>T	p.Glu1113X	Exon 25	-	N/A	-	-	This study
	Missense	c.3724G>A	p.Val1242Met	Exon 28	Cadherin 11	0	0	1	This study

Computer analysis to predict the effect of missense variants on MYO7A protein function was performed with sorting intolerant from tolerant (SIFT; http://sift.jcvi.org/), and polymorphism phenotyping (PolyPhen2; http://genetics.bwh.harvard.edu/pph2/).

N/A: not applicable.

All mutations were detected in only one patient each and sixteen of the 17 patients (94.1%) carried at least one mutation, while one patient had no mutations. Thirteen of the 16 mutation carriers each had two pathogenic mutations (Table 2).

**Table 2:** Details of phenotype and genotype of 17 USH1 patients.

Sample No.	Age	Sex	Allele1	Allele2	Hereditary form	Onset of night blindness	Cataract	Hearing Aid	Cochlear Implant
***MYO7A***									
1	37	M	p.Gln493X	p.Trp1495CysfsX55	sporadic	13	no	unilateral	unilateral
2	41	W	p.l2069fsX6	p.l2069fsX6	AR	unknown	both eyes	bilateral	no
5	54	M	p.Val692Met	p.Val692Met	AR	5	both eyes	no	no
6	54	W	p.Arg570X	p.Arg570X	sporadic	6	no	no	no
8	14	M	p.Lys542GlnfsX5	p.Lys542GlnfsX5	sporadic	6	no	unilateral	unilateral
11	54	M	p.Asp2010Asn	p.Trp2107X	sporadic	13	no	no	no
17	56	W	p.Cys705X	p.Cys705X	sporadic	unknown	no	no	no
***CDH23***									
7	12	W	p.Arg1189ArglfsX5	p.Arg1189ArglfsX5	sporadic	12	both eyes	no	bilateral
9	9	M	p.Ser1927Cysfs16	c.5821-2A>G	sporadic	8	no	unilateral	unilateral
15	16	W	p.Arg2107X	p.Arg2107X	sporadic	unknown	no	no	no
***PCDH15***									
3	47	W	p.Glu1113X	p.Glu1113X	sporadic	5	both eyes	no	no
16	28	W	p.Arg991X	p.Arg991X	AR	10	no	no	no
10	62	M	p.Arg962Cys	unknown	sporadic	9	both eyes	no	no
12	52	M	p.Arg336X	unknown	sporadic	3	no	no	no
13	51	M	p.Val1242Met	unknown	sporadic	10	no	no	no
***MYO7A*** * **^1^** ***/PCDH15*** * **^2^**									
4	21	M	p.Ala771Ser* ^1^	c.158-1G>A* ^2^	sporadic	10	no	unilateral	unilateral
**unknown**									
14	64	W	unknown	unknown	sporadic	15	both eyes	unilateral	no

*All subjects have congenital deafness and RP.

Nonsense, frame shift, and splice site mutations are all classified as pathogenic, whereas missense mutations are presumed to be probable pathogenic variants based on results of prediction software for evaluation of the pathogenicity of missense variants (Table 1).

Of the 19 probable pathogenic mutations that we found, 17 were detected by MPS. The remaining two (p.Lys542GlnfsX5 in *MYO7A* and c.5821-2A>G in *CDH23*) were sequenced by direct sequence analysis.

Of our 17 USH patients, seven had *MYO7A* mutations (41.2%), three had *CDH23* mutations (17.6%), and two had *PCDH15* mutations (11.8%). We did not find any probable pathogenic mutations in *USH1C*, *USH1G*, and USH2/3 genes.

**Figure 1:**
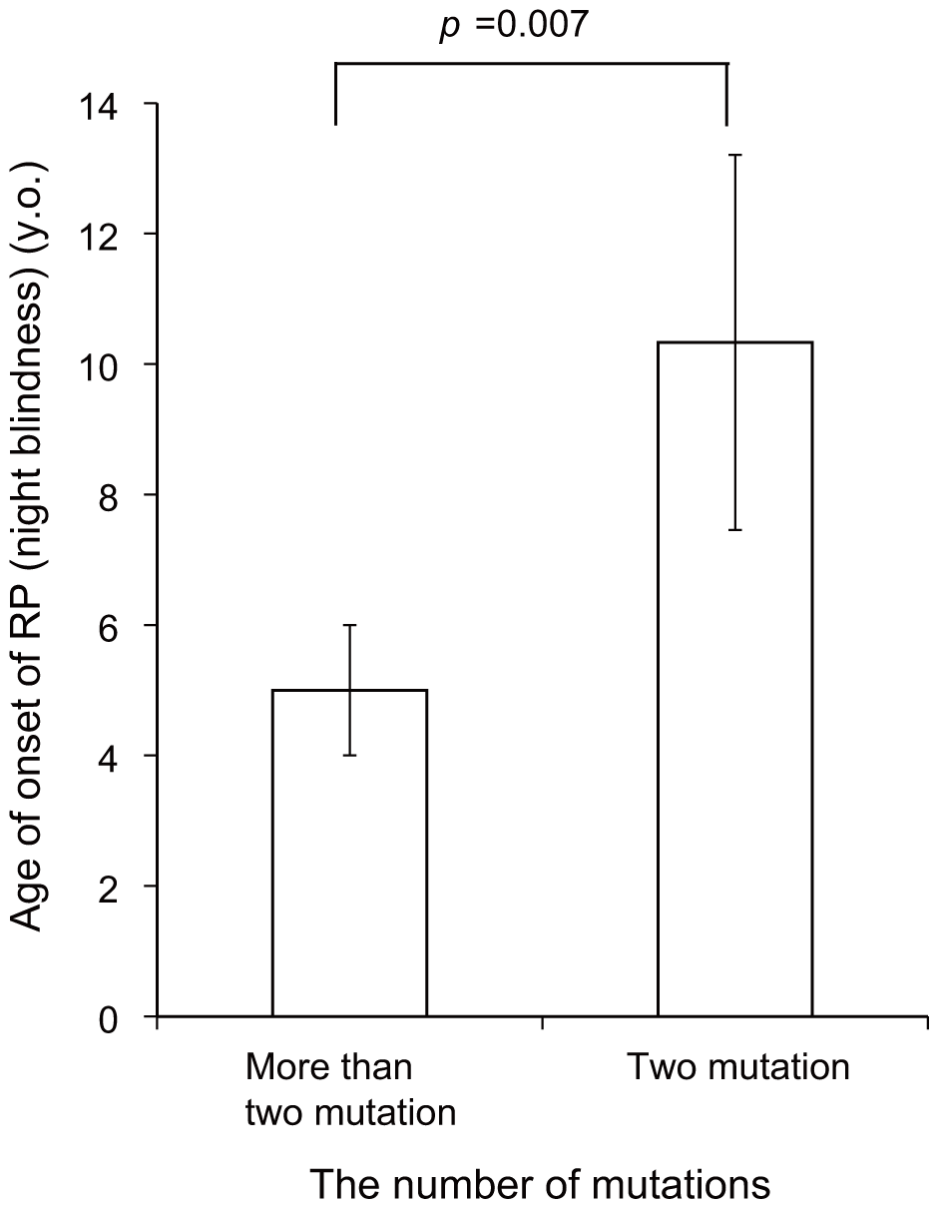
The number of mutations and the age of RP onset in Usher syndrome type 1 patients. The age of RP onset is earlier in the patients with more than two pathogenic mutations. RP: retinitis pigmentosa.

Four USH1 patients (Cases #3, 5, 8, 15) had probable pathogenic mutations in two different USH genes, with one being a biallelic mutation (Table 3). The other heterozygous/homozygous mutations were missense variants. Three of these patients (Cases #3, 5, 8) presented with earlier RP onset (night blindness) than in the other patients with two pathogenic mutations (Cases #1, 6, 7, 9, 11, 16) (*p* = 0.007) (Fig. 1).

**Table 3:** The patients with mutations in two different genes.

Sample	Genes with two pathogenic mutations	Gene with one heterozygous mutation	Nucleotide change	Amino acid change	control	SIFT score	PolyPhen score	Referense
5	*MYO7A*	*CDH23*	c.C719T	p.P240L*	0.26	0.06	0.999	Wagatsuma et al. (2007)
8	*MYO7A*	*CDH23*	c.2568C>G	p.Ile856Met	0	0.08	1	This study
15	*CDH15*	*USH1C*	c.2437T>G	p.Tyr813Asp	0	0.19	0.932	This study
3	*PCDH15*	*USH1G*	c.28C>T	p.Arg10Trp	0	0.19	1	This study

*homozygotes.

**Figure 2:**
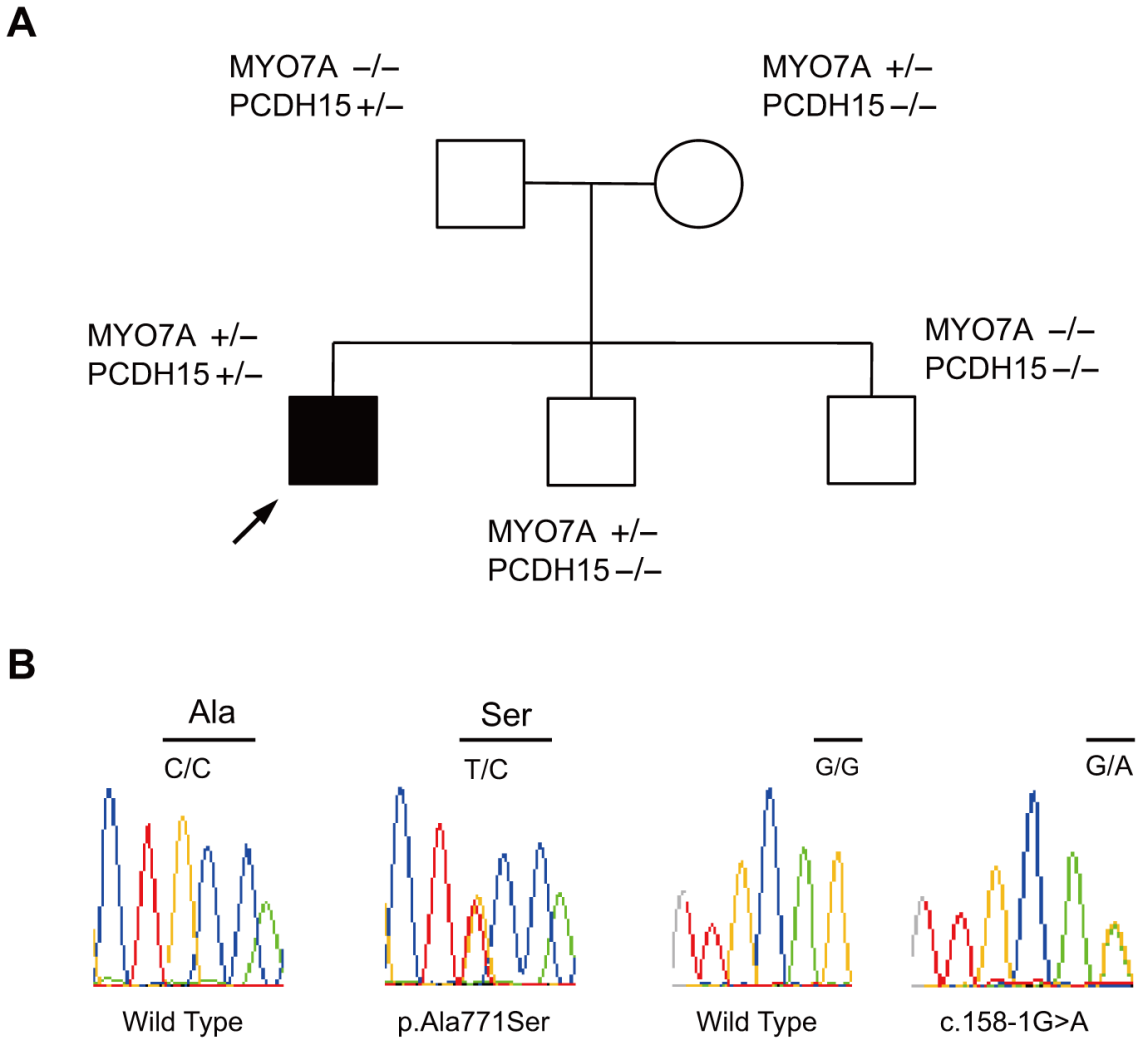
Pedigree and sequence chromatograms of the patient with the p.Ala771Ser in *MYO7A* and c.158-1G>A in *PCDH15* mutations. (A) The pedigree and sequence results of the proband and family. (B) Sequence chromatograms from wild-type and mutations. The proband, his mothor and one brother carried a heterozygous 2311G>T transition in exon 20, which results in an alanine to a serine (Ala771Ser) in *MYO7A*. Another variation, 158-1G>A in intron 3 of *PCDH15*, was derived from the proband and his father. Another brother had no variants.

One patient (Case #4) had heterozygote mutations in two USH1 genes (p.Ala771Ser in *MYO7A* and c.158-1G>A in *PCDH15*). His parents and one brother were found to also be carriers for these mutations. Another brother had no variants (Fig. 2).

## Discussion

For USH1, early diagnosis has many immediate and several long-term advantages for patients and their families [1]. However, diagnosis in childhood, based on a clinical phenotype, has been difficult because patients appear to have only non-syndromic HL in childhood and RP develops in later years. Although early diagnosis is now possible through DNA testing, performing large-scale mutation screening for USH genes in all non-syndromic HL children has been both time-consuming and expensive. Therefore, the availability of MPS, which facilitates comprehensive large-scale mutation screening [19] is a very welcome advance.

MPS technology enabled us to detect pathogenic mutations in USH1 patients efficiently, identifying one or two pathogenic/likely pathogenic mutations in 16 of 17 (94.1%) cases. This was comparable to previous direct sequence analysis results such as Bonnet et al. who detected one or two mutations in 24 out of 27 (89%) USH1 patients [11] and Le Quesne Stabej et al. who detected one or two mutations in 41 out of 47 (87.2%) USH1 patients [12].

In addition, MPS assists in the analysis of disease modifiers and digenic inheritance because it simultaneously investigates many causative genes for a specific disease, such as in our case, USH. Previous reports have described several USH cases with pathogenic mutations in two or three different USH genes [11], [12], [20]. In our study, four patients had two pathogenic mutations in one gene and missense variants in a different gene (Table 3). We considered the latter to possibly be a disease modifier. For example, *USH1C*:p.Tyr813Asp, which occurred in 0/384 control chromosomes and was predicted to be “probably damaging” by the Polyphen program, was found with a homozygous *CDH23* nonsense mutation (p.Arg2107X) (Case #15). As for what the variant “modifies”, we speculate that for USH1 patients with a disease modifier, RP symptoms such as night blindness show an earlier onset. However, we think that profound HL and the absence of vestibular function in USH1 patients are not affected by modifiers as they are congenital and therefore not progressive.

Ebermann et al. described a USH2 patient with “digenic inheritance.” a heterozygous truncating mutation in *GPR98*, and a truncating heterozygous mutation in PDZ domain-containing 7 (*PDZD7*), which is reported to be a cause of USH [20]. Our USH1 patient (Case #4) had segregated *MYO7A*:p.Ala771Ser and *PCDH15*:c.158-1G>A. Molecular analyses in mouse models have shown many interactions among the USH1 proteins [2]. In particular, *MYO7A* directly binds to *PCDH15* and both proteins are expressed in an overlapping pattern in hair bundles in a mouse model [21]. *PCDH15*:c.158-1G>A, predicted to alter the splice donor site of intron 3, has been classified as pathogenic. *MYO7A*:p.Ala771Ser is a non-truncating mutation, but was previously reported as disease-causing [13]. So, we consider the patient to be the first reported case of *MYO7A*/*PCDH15* digenic inheritance.

However, we should be aware of two limitations of MPS technology. First, the target region of MPS cannot cover all coding exons of USH genes. Actually, the coverage of the target exons was 97.0% in our study. So, it is impossible to detect a mutation in a region which is not covered using this system (Case #9: c.5821-2A>G). Secondarily, the MPS system used in this study, is not effective for detecting homo-polymer regions, for example poly C stretch [22] (Case #8: p.Lys542GlnfsX5). In addition, concerning pathogenecity of mutations identified, functional analysis will be necessary to draw the final conclusion in the future.

In UK and US Caucasian USH1 patients, USH1B (*MYO7A*) has been reported as the most common USH1 genetic subtype [11], [12], while USH1F (*PCDH15*) has been reported as the most common USH1 genetic subtype in North American Ashkenazi Jews [23]. In Japanese, our study revealed that the most common type was *MYO7A* (41.7%), which was similar to the frequency in the above Caucasian patients (46.8∼55%) [11], [12]. However, the small number of USH1 patients in our study might have biased the frequency and further large cohort study will be needed in the future.

In addition, most of our detected mutations were novel. We have previously reported genes responsible for deafness in Japanese patients and observed differences in mutation spectrum between Japanese (who are probably representative of other Asian populations) and populations with European ancestry [24].

In conclusion, our study was the first report of USH mutation analysis using MPS and the frequency of USH1 genes in Japanese. Mutation screening using MPS has the potential power to quickly identify mutations of many causative genes such as USH while maintaining cost-benefit performance. In addition, the simultaneous mutation analysis of large numbers of genes was useful for detecting mutations in different genes that are possibly disease modifiers or of digenic inheritance.

## Materials and Methods

### Subjects

We screened 17 Japanese USH1 patients (aged 9 to 64 years): three from autosomal recessive families (non-affected parents and two or more affected siblings), and 14 from sporadic families. There were 9 males and 8 females. None of the subjects had any other noteworthy symptoms. All subjects or next of kin on the behalf of the minors/children gave prior written informed consent for participation in the project, and the Ethical Committee of Shinshu University approved the study and the consent procedure.

### Amplicon Library Preparation

An Amplicon library of the target exons was prepared with an Ion AmpliSeq Custom Panel (Applied Biosystems, Life Technologies, Carlsbad, CA) designed with Ion AmpliSeq Designer (https://www.ampliseq.com/browse.action) for nine USH genes by using Ion AmpliSeq Library Kit 2.0 (Applied Biosystems, Life Technologies) and Ion Xpress Barcode Adapter 1–16 Kit (Applied Biosystems, Life Technologies) according to the manufacturers' procedures.

In brief, DNA concentration was measured with Quant-iT dsDNA HS Assay (Invitrogen, Life Technologies) and Qubit Fluorometer (Invitrogen, Life Technologies) and DNA quality was confirmed by agarose gel electrophoresis. 10 ng of each genomic DNA sample was amplified, using Ion AmpliSeq HiFi Master Mix (Applied Biosystems, Life Technologies) and AmpliSeq Custom primer pools, for 2 min at 99°C, followed by 15 two-step cycles of 99°C for 15 sec and 60°C for 4 min, ending with a holding period at 10°C in a PCR thermal cycler (Takara, Shiga, Japan). After the Multiplex PCR amplification, amplified DNA samples were digested with FuPa enzyme at 50°C for 10 min and 55°C for 10 min and the enzyme was successively inactivated for 60°C for 20 min incubation. After digestion, diluted barcode adapter mix including Ion Xpress Barcode Adapter and Ion P1 adaptor were ligated to the end of the digested amplicons with ligase in the kit for 30 min at 22°C and the ligase was successively inactivated at 60°C for 20 min incubation. Adaptor ligated amplicon libraries were purified with the Agencourt AMPure XP system (Beckman Coulter Genomics, Danvers, MA). The amplicon libraries were quantified by using Ion Library Quantitation Kit (Applied Biosystems, Life Technologies) and the StepOne plus realtime PCR system (Applied Biosystems, Life Technologies) according to the manufacturers' procedures. After quantification, each amplicon library was diluted to 20 pM and the same amount of the 12 libraries for 12 patients were pooled for one sequence reaction.

### Emulsion PCR and Sequencing

The emulsion PCR was carried out with the Ion OneTouch System and Ion OneTouch 200 Template Kit v2 (Life Technologies) according to the manufacturer's procedure (Publication Part Number 4478371 Rev. B Revision Date 13 June 2012). After the emulsion PCR, template-positive Ion Sphere Particles were enriched with the Dynabeads MyOne Streptavidin C1 Beads (Life Technologies) and washed with Ion OneTouch Wash Solution in the kit. This process were performed using an Ion OneTouch ES system (Life Technologies).

After the Ion Sphere Particle preparation, MPS was performed with an Ion Torrent Personal Genome Machine (PGM) system using the Ion PGM 200 Sequencing Kit and Ion 318 Chip (Life Technologies) according to the manufacturer's procedures.

### Base Call and Data Analysis

The sequence data were processed with standard Ion Torrent Suite Software and Torrent Server successively mapped to human genome sequence (build GRCh37/hg19) with Torrent Mapping Alignment Program optimized to Ion Torrent data. The average of 562.33 Mb sequences with about 4,300,000 reads was obtained by one Ion 318 chip. The 98.0% sequences were mapped to the human genome and 94% of them were on the target region. Average coverage of depth in the target region was 314.2 and 93.8% of them were over 20 coverage.

After the sequence mapping, the DNA variant regions were piled up with Torrent Variant Caller plug-in software. Selected variant candidates were filtered with the average base QV (minimum average base quality 25), variant frequency (40–60% for heterozygous mutations and 80–100% for homozygous mutations) and coverage of depth (minimum coverage of depth 10). After the filtrations, variant effects were analyzed with the wANNOVAR web site [25], [26] (http://wannovar.usc.edu) including the functional prediction software for missense variants: Sorting Intolerant from Tolerant (SIFT; http://sift.jcvi.org/), and Polymorphism Phenotyping (PolyPhen2; http://genetics.bwh.harvard.edu/pph2/). The sequencing data was available in the DNA databank of Japan (Accession number: DRA001273).

### Algorithm

Missense, nonsense, and splicing variants were selected among the identified variants. Variants were further selected as less than 1% of: 1) the 1000 genome database (http://www.1000genomes.org/), 2) the 5400 exome variants (http://evs.gs.washington.edu/EVS/), and 3) the in-house control. Candidate mutations were confirmed by Sanger sequencing and the responsible mutations were identified by segregation analysis using samples from family members of the patients. In addition, the cases with heterozygous or no causative mutation were fully sequenced by Sanger sequencing for USH1 genes in order to verify the MPS results.

### Direct Sequence Analysis

Primers were designed with the Primer 3 plus web server (http://www.bioinformatics.nl/cgi-bin/primer3plus/primer3plus.cgi). Each genomic DNA sample (40 ng) was amplified using Ampli Taq Gold (Life Technologies) for 5 min at 94°C, followed by 30 three-step cycles of 94°C for 30 sec, 60°C for 30 sec, and 72°C for 30 sec, with a final extension at 72°C for 5 min, ending with a holding period at 4°C in a PCR thermal cycler (Takara, Shiga, Japan). The PCR products were treated with ExoSAP-IT (GE Healthcare Bio, Buckinghamshire, UK) and by incubation at 37°C for 60 min, and inactivation at 80°C for 15 min. After the products were purified, we performed standard cycle sequencing reaction with ABI Big Dye terminators in an ABI 3130xl sequencer (Life Technologies).

### Accession numbers

*MYO7A*, [NM_000260.3]; *USH1C*, [NM_ 153676.3]; *CDH23*, [NM_ 022124.5]; *PCDH15*, [NM_ 033056.3]; *USH1G*, [NM_ 173477.2]; *USH2A*, [NM_206933.2]; *GPR98*, [NM_ 032119.3]; *DFNB31*, [NM_ 015404.3]; *CLRN1*, [NM_ 174878.2]; *PDZD7*, [NM_ 001195263.1].
